# Definitive Local Therapy Is Associated with Improved Survival in Metastatic Soft Tissue Sarcomas

**DOI:** 10.3390/cancers13050932

**Published:** 2021-02-24

**Authors:** Vishruth K. Reddy, Varsha Jain, Sriram Venigalla, Vivek Nimgaokar, Ashwin Amurthur, Daniel Y. Lee, Ronnie A. Sebro, Robert G. Maki, Robert J. Wilson, Kristy L. Weber, Jacob E. Shabason

**Affiliations:** 1 Department of Radiation Oncology, University of Pennsylvania, Philadelphia, PA 19104, USA; vishruth.reddy@pennmedicine.upenn.edu (V.K.R.); varsha.jain@jefferson.edu (V.J.); sriram.venigalla@pennmedicine.upenn.edu (S.V.); 2 Perelman School of Medicine, University of Pennsylvania, Philadelphia, PA 19104, USA; vivek.nimgaonkar@pennmedicine.upenn.edu (V.N.); ashwin.amurthur@pennmedicine.upenn.edu (A.A.); danielyounghoon.lee@pennmedicine.upenn.edu (D.Y.L.); 3 Department of Biostatistics, Epidemiology and Bioinformatics, University of Pennsylvania, Philadelphia, PA 19104, USA; ronnie.sebro@pennmedicine.upenn.edu; 4 Department of Genetics, University of Pennsylvania, Philadelphia, PA 19104, USA; 5 Department of Radiology, University of Pennsylvania, Philadelphia, PA 19104, USA; 6 Department of Orthopedic Surgery, University of Pennsylvania, Philadelphia, PA 19104, USA; robert.wilson3@pennmedicine.upenn.edu (R.J.W.II); kristy.weber@pennmedicine.upenn.edu (K.L.W.); 7 Division of Hematology/Oncology, Department of Medicine, University of Pennsylvania, Philadelphia, PA 19104, USA; robert.maki@pennmedicine.upenn.edu

**Keywords:** sarcoma, radiation, surgery, local treatment, survival

## Abstract

**Simple Summary:**

Patients with metastatic soft tissue sarcomas (STS) often receive definitive local treatment with surgery and/or radiation in addition to chemotherapy to reduce morbidity associated with local tumor progression. We hypothesized that definitive local treatment is associated with improved overall survival (OS). We utilized the National Cancer Database to assess the association between definitive local treatment and OS, and factors associated with the receipt of definitive local therapy. Compared with chemotherapy alone, receipt of any definitive local therapy was associated with improved OS (median 17.9 vs. 10.1 months). The survival benefit remained on multivariate analyses and propensity-score matched analyses, with a stepwise improvement with surgery and combined modality local therapy, specifically radiotherapy (HR: 0.77; *p* < 0.001), surgery (HR: 0.67; *p* < 0.001), and combined surgery and radiotherapy (HR: 0.42; *p* < 0.001). Our study suggests that chemotherapy plus definitive local treatment is associated with a significant survival benefit compared to the standard chemotherapy alone for patients with metastatic STS.

**Abstract:**

*Background:* Definitive local therapy is often utilized in patients with metastatic soft tissue sarcomas (STS) to reduce morbidity associated with local tumor progression. We hypothesize that it is associated with improved overall survival (OS). *Methods:* Patients with newly diagnosed metastatic STS treated with chemotherapy were identified from the National Cancer Database and dichotomized into cohorts: 1. definitive local therapy (defined as either definitive dose radiotherapy, definitive surgery, or surgery with perioperative radiotherapy) or 2. conservative therapy (defined as systemic therapy with or without palliative therapy). The association between definitive local therapy and OS, and factors associated with the receipt of definitive local therapy were assessed. *Results:* Total of 4180 patients were identified. Compared with the conservative therapy, receipt of any definitive local therapy was associated with improved OS (median 17.9 vs. 10.1 months). The survival benefit remained on multivariate analyses and propensity-score matched analyses, with a stepwise improvement with surgery and combined modality local therapy, specifically radiotherapy (HR: 0.77; *p* < 0.001), surgery (HR: 0.67; *p* < 0.001), and combined surgery and radiotherapy (HR: 0.42; *p* < 0.001). *Conclusions:* Analysis of a large national cancer registry of patients with metastatic STS suggests that chemotherapy plus definitive local therapy is associated with a significant survival benefit compared to the standard chemotherapy alone.

## 1. Introduction

Over 13,000 patients are diagnosed with soft tissue sarcomas (STS) in the United States every year, with approximately 15% found to have metastatic disease at the time of diagnosis [1,2]. The mainstay of treatment for those with metastatic disease at diagnosis is chemotherapy, with local therapy often used for palliation of symptoms [3]. However, despite advances in systemic therapeutic agents for this patient population, the overall prognosis remains poor, with an estimated 5-year overall survival (OS) of 16% [2], and a median OS for those treated with systemic therapy estimated to be between 12 and 17 months [4,5]. An open question remains if improved local control has the potential to impact these outcomes.

Given the discordant evidence as to the potential benefits of locally directed therapy in those with metastatic disease [6,7,8,9], latest guidelines reflect that there is no standard, optimal management for these patients [3]. However, systemic therapy alone is frequently insufficient to mitigate progression of the disease at the primary site and corresponding morbidity [6]. Numerous retrospective and prospective studies of patients with metastatic disease across various malignancies have suggested possible survival benefits of definitive local therapy to the primary site [10,11,12,13,14,15,16,17,18]. As such, we sought to evaluate the potential survival benefit of definitive local therapy in patients with metastatic STS. Using the National Cancer Database (NCDB), we examined the patterns of use and survival outcomes associated with definitive local therapy in patients with newly diagnosed metastatic STS who also received chemotherapy. We hypothesized that chemotherapy with definitive local therapy is associated with improved survival compared to chemotherapy alone.

## 2. Results

### 2.1. Baseline Clinical Characteristics

**Figure 1:**
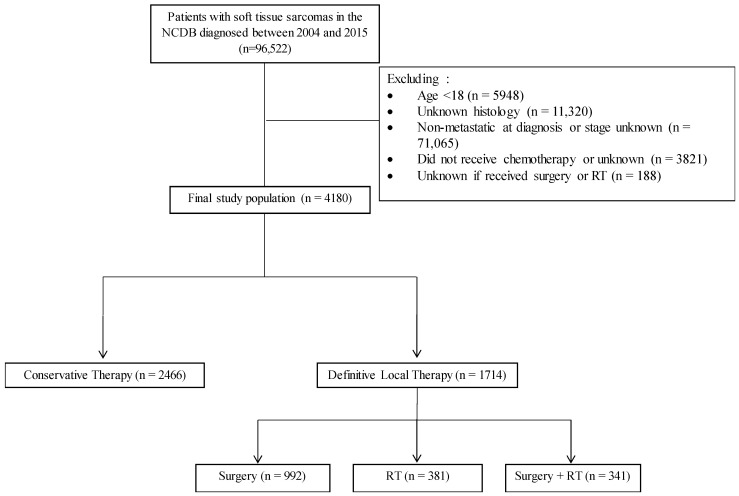
Consolidated standards of reporting trials (CONSORT) diagram of the patient cohort. NCDB = National Cancer Database. RT = radiotherapy.

A total of 4180 patients met the study inclusion criteria (Figure 1). Complete patient characteristics are shown in the Table S1. Notably, the median age of the patient cohort was 56 years (range, 18–90 years). The majority of patients were men (55%), Non-Hispanic white (72%), and had tumors arising from the extremity (37%). The majority of patients were treated with conservative therapy (59%) rather than definitive local therapy (41%). Of those treated with definitive local therapy, 58% were treated with surgery, 22% with radiotherapy, and 20% with combined surgery and perioperative radiation.

### 2.2. Factors Associated with Receipt of Definitive Local Therapy

On multivariable analysis, notable sociodemographic predictors of omission of definitive therapy included Medicare insurance (OR: 0.78, 95% CI 0.64–0.95, *p* < 0.05) or no insurance (OR: 0.66, 95% CI 0.49–0.88, *p* < 0.01) (Table 1). Likewise, clinical predictors of omission of definitive local therapy included those with thoracic (OR: 0.55, 95% CI 0.44–0.69, *p* < 0.001) or abdominal/pelvic tumors (OR: 0.49, 95% CI 0.42–0.57, *p* < 0.001) (Table 1). Treatment at an academic institution (OR: 1.17, 95% CI 1.00–1.37, *p* = 0.05) was associated with receipt of definitive local therapy. Additional factors associated with the modality of local therapy are described in the Tables S2–S4.

### 2.3. Impact of Definitive Local Therapy on Overall Survival

**Figure 2:**
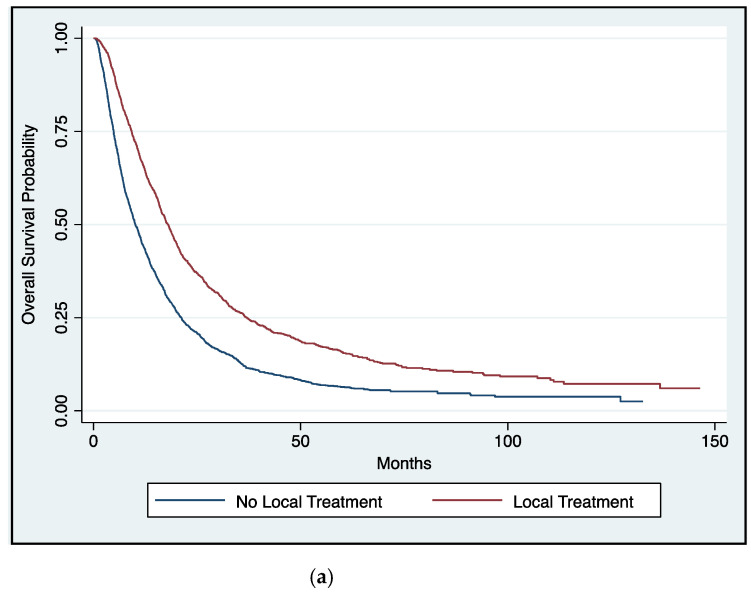

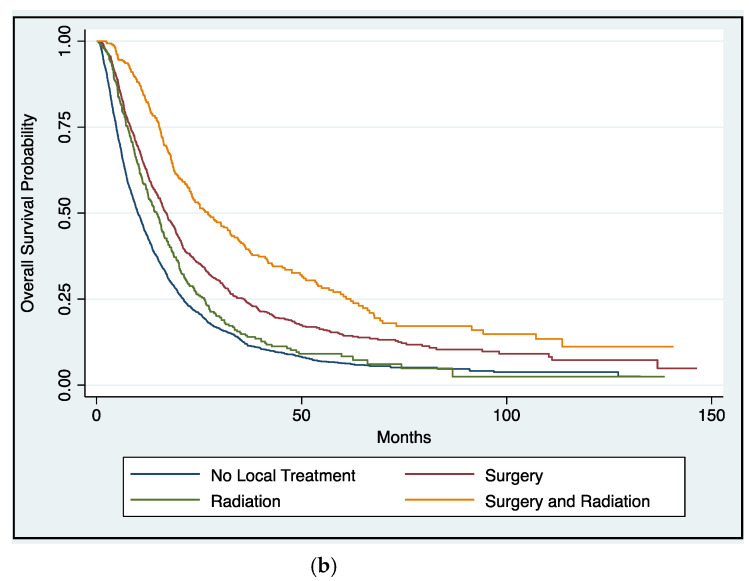
(**a**): Overall survival as a function of receipt of definitive local therapy vs. none in patients with metastatic sarcoma (log rank *p* < 0.001). (**b**): Overall survival as a function of receipt of definitive local therapy in patients with metastatic sarcoma (log rank *p* < 0.001).

Compared with conservative therapy, receipt of any definitive local therapy was associated with improved OS with a median survival of 17.9 vs. 10.1 months and a 5-year OS rate of 15.8% vs. 6.4% (*p* < 0.001) (Figure 2a). There appeared to be a stepwise improvement in survival outcomes with more aggressive local therapy with a median survival of 14.7 months, 17.0 months, and 27.2 months for radiation, surgery, and surgery plus perioperative radiation, respectively (*p* < 0.001) (Figure 2b). The survival benefit of definitive local therapy remained on multivariate analyses with a stepwise improvement with surgery and combined modality local therapy, specifically radiation (HR: 0.77; 95% CI, 0.67–0.87; *p* < 0.001), surgery (HR: 0.67; 95% CI, 0.61–0.73; *p* < 0.001), and combined surgery and radiotherapy (HR: 0.42; 95% CI, 0.36–0.48; *p* < 0.001) (Table 2).

**Figure 3:**
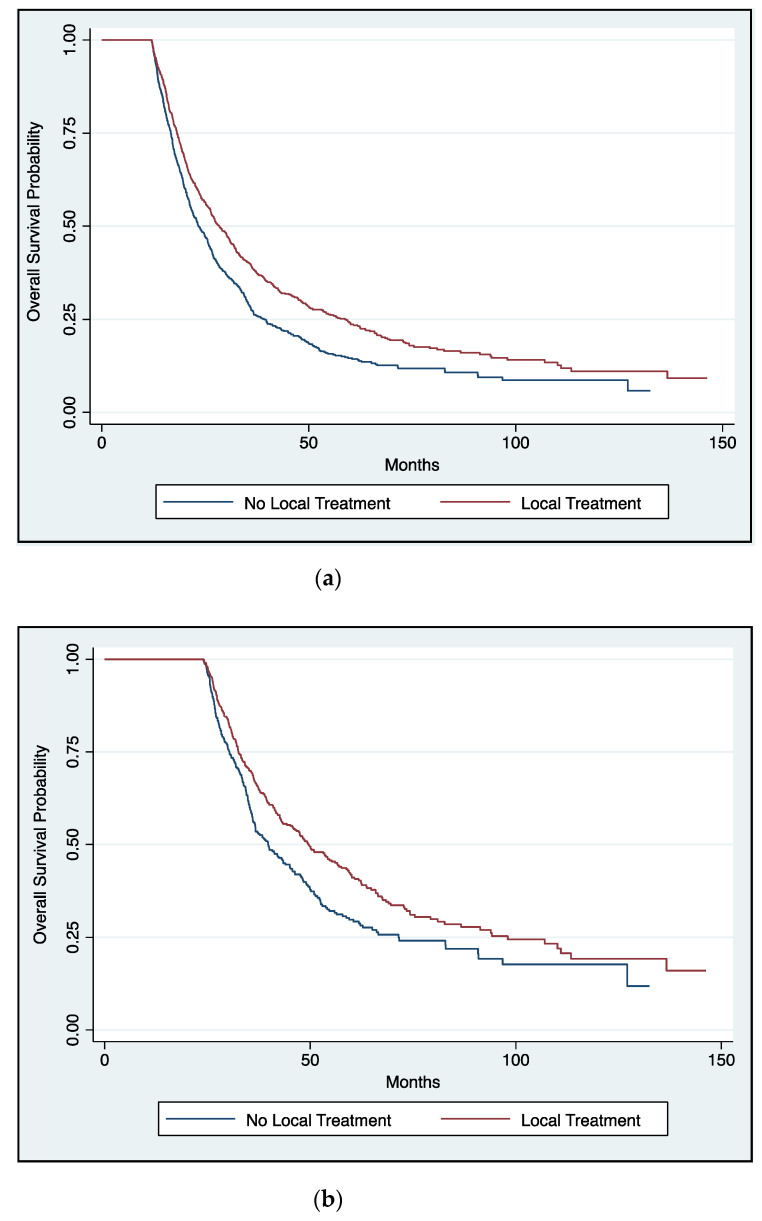
(**a**): Overall survival as a function of receipt of definitive local therapy vs. none in patients with metastatic sarcoma, those surviving >12 months (log rank *p* < 0.001). (**b**): Overall survival as a function of receipt of definitive local therapy vs. none in patients with metastatic sarcoma, those surviving >24 months (log rank *p* < 0.001).

The improvement in OS remained after PS analysis for radiation (HR: 0.75; 95% CI, 0.66–0.86; *p* < 0.001), surgery (HR: 0.66; 95% CI, 0.60–0.72; *p* < 0.001), and combined surgery and radiotherapy (HR: 0.41; 95% CI, 0.35–0.47; *p* < 0.001) (Table 2). Furthermore, this survival benefit was retained on landmark analyses at 12 months (*p* < 0.001) (Figure 3a) and 24 months (*p* < 0.001) (Figure 3b), suggesting they were unaffected by immortal time biases.

## 3. Discussion

We utilized a national cancer registry to evaluate the benefits of definitive local therapy in patients with metastatic STS. In our study we demonstrate an association between definitive local therapy and improved OS when compared with the conservative therapy alone in over 4000 patients with newly diagnosed metastatic STS. Furthermore, there appears to be an incremental survival benefit for the more aggressive local therapy. To our knowledge, this is the most comprehensive study to examine the benefits of definitive local therapy in this population.

National guidelines reflect that there is no standard, optimal management for patients with metastatic STS [3] given the discrepant results as to the potential benefits of locally-directed therapy [6,7,8,9]. However, the challenge remains that despite the advances in systemic therapy in recent years, these changes have translated into only incremental survival improvements, and the prognosis of this population remains poor with an estimated 5-year OS of 16% [2,6]. Recently, there has been considerable interest in the potential benefits of definitive rather than palliative local therapy to the primary site even in the metastatic setting across a number of disease sites with both retrospective and prospective data demonstrating improvement in progression and overall survival for patients [10,11,12,13,14,15,16,17,18]. There have been many theories as to the mechanism of improvement in survival with the treatment of local disease, ranging from prevention of life-threatening complications from local progression to potential decreased risk of subsequent metastatic seeding through better control of the primary site of disease [19]. Mechanistic studies via animal models have suggested that the benefits of primary site cytoreduction with definitive treatment may be due to a reversal of tumor-associated immunosuppression and corresponding improvement in the host immune defenses directed at the metastatic sites of disease [20].

While we demonstrate a benefit associated with definitive local therapy for metastatic STS, it is important to highlight that such therapy is likely not the optimal treatment option for all patients with metastatic disease. Indeed, this treatment may be best employed in a carefully selected group of patients with good performance status and a low-to-moderate metastatic disease burden. Therefore, careful selection in regards to aggressive therapy is vital, particularly in patients with incurable disease, as potential for overtreatment exists and can certainly be harmful.

Strengths of the present study include a modern cohort of patients treated for STS and adjustment for a range of patient- and facility-level variables. Our study has several notable limitations given its retrospective design and reliance on the content and accuracy of information included in the NCDB. Additionally, there is inherent selection bias associated with the retrospective nature of this analysis. It is also possible that we were unable to account for the several unmeasured confounders such as patient preferences, physician attitudes, referral patterns, and quality of care received, which impacted patient selection and management. Importantly, we cannot determine the burden of metastatic disease or initial response to systemic therapy. These clinical factors certainly had a major impact on which patients were deemed appropriate for definitive local therapy. Despite these limitations, however, we aimed to more robustly account for the baseline difference between cohorts with propensity score matching, with our results demonstrating that the survival benefit associated with definitive local therapy remained. Additionally, our study suggest that this benefit was also unaffected by immortal time biases as evidenced by the results of our landmark analysis of patients who survived at least 24 months.

## 4. Materials and Methods

### 4.1. Data Source

The study population was identified from the National Cancer Database (NCDB), a national cancer registry jointly sponsored by the American College of Surgeons and the American Cancer Society that draws upon hospital registry data from more than 1500 Commission on Cancer (CoC)-accredited facilities in the United States [21,22]. The dataset captures more than 70% of incident cancers and comprises more than 34 million unique cancer cases [21,22]. Data are collected prospectively from Commission on Cancer—accredited program cancer registries with nationally standardized data-coding definitions.

### 4.2. Study Population

Inclusion criteria (Figure 1) for the cohort consisted of patients ≥ 18 years with newly diagnosed metastatic STS treated with systemic therapy, which remains the most common approach in the metastatic setting [3]. Metastatic disease was defined as per the American Joint Committee on Cancer staging system definition of M1 disease. Patients could have received local therapy with either radiation therapy, surgery, or surgery with perioperative radiation which reflects guideline-based suggestions for patients amenable to or requiring local therapy [3]. Patients with STS arising in the head, neck, extremities, thorax, trunk, abdomen, and pelvis and those with common adult STS histologies such as undifferentiated or unclassified histology, undifferentiated pleomorphic sarcoma, liposarcoma, leiomyosarcoma, fibrosarcoma, synovial sarcoma, angiosarcoma, and malignant peripheral nerve sheath tumor were included. Gastrointestinal stromal tumors were not included.

### 4.3. Patient Cohorts and Variables

The overall patient cohort was dichotomized into definitive local therapy and conservative therapy cohorts. Definitive local therapy was defined as the receipt of either surgery, definitive radiation to >4400 cGy, or combined modality surgery and radiotherapy in addition to systemic therapy. Conservative therapy was defined as the receipt of systemic therapy with or without a palliative dose (≤4400 cGy) of radiation. The covariates examined included sex, age, race, population density of patient residence (classified as metropolitan, urban, or rural from data published by the USDA Economic Research Service), facility geographic location, facility type (nonacademic or academic), with academic referring to academic/research programs including NCI-designated comprehensive cancer centers), distance to treatment facility (calculated by distance between patient’s zip code center and hospital street address), educational attainment (defined as percentage of population in patient’s ZIP code without a high school degree, which is derived from the US Census data), income (defined as the median income in patient’s ZIP code), Charlson/Deyo comorbidity score (a validated, weighted measure of comorbidity of patients) [23], primary site of tumor, tumor size, tumor grade, and year of treatment.

### 4.4. Endpoints

The primary endpoint was OS, which was defined as the time from the date of the initial diagnosis until death or last follow-up. We also assessed the patterns of use of definitive local therapy relative to conservative therapy.

### 4.5. Statistical Analysis

Baseline characteristics of the study cohorts were compared using Pearson’s chi-squared tests. All covariates achieving a threshold significance of *p* < 0.1 on univariate analysis were included in the multivariable logistic regression model to assess the independent effect of each covariate on the odds of being treated with definitive local therapy relative to conservative therapy. A Cox proportional hazards regression model was created to assess the independent effect of definitive local therapy on OS compared with conservative therapy. The Kaplan–Meier estimator and log-rank test were used to compare OS between the cohorts. To more robustly account for baseline difference between cohorts, a secondary survival analysis was performed using propensity score (PS) matched cohorts for those treated with definitive local therapy. Those treated with definitive local therapy were matched to those treated with conservative therapy. This was done using 1-to-1 nearest neighbor matching without replacement [24] (matched for all covariates listed in Table 2). Absolute standardized differences of <0.1 between baseline covariates following matching were accepted as a measure of adequate balance [25]. A Cox survival analysis was then repeated on the matched cohorts to estimate the hazard of death associated with receipt of definitive local therapy. A landmark analysis [26] of patients who survived at least 24 months was conducted to account for immortal time bias. A two-tailed *p*-value < 0.05 was considered statistically significant. Statistical analyses were performed using Stata SE, version 15.0 (StataCorp, College Station, TX, USA).

## 5. Conclusions

In conclusion, our results demonstrate an association between definitive local therapy and improved OS in a cohort of patients with newly diagnosed metastatic STS. This study adds to a growing body of literature across numerous cancer sites in support of the selective use of definitive local therapy as a potentially beneficial therapeutic strategy in the setting of metastatic disease. It highlights that local control should be carefully considered, while understanding that patient comorbidities may play an equal, if not more important, role in the ultimate decision of how to manage these patients. Indeed, in our patient cohort, the majority of patients (59%) did not receive definitive local treatment, perhaps because certain clinical factors or comorbidities rendered them suboptimal candidates and because of conflicting prior data with regards to the potential benefits of local treatment. It is important to consider that definitive local therapy can certainly come with treatment toxicity and impact patients’ quality of life. Prospective validation of these findings is warranted to help establish optimal patient selection for definitive therapy and potentially improve outcomes for those with metastatic STS.

## Figures and Tables

**Table 1:** Factors associated with receipt of definitive local therapy. Univariate analysis and multivariate analysis, respectively.

Receipt of Local Therapy	OR (95% CI)	*p* Value	OR (95% CI)	*p* Value
**Age**				
<70 years	1		1	
≥70 years	0.74 (0.63–0.87)	<0.001	0.90 (0.72–1.12)	0.326
**Gender**				
Male	1		---	---
Female	0.99 (0.87–1.12)	0.853	---	---
**Race**				
Non-Hispanic White	1		---	---
Non-Hispanic Black	0.91 (0.76–1.08)	0.291	---	---
Hispanic	0.95 (0.76–1.19)	0.645	---	---
Other	0.95 (0.70–1.27)	0.720	---	---
**Facility Area**				
Metropolitan	1		---	---
Urban	0.97 (0.80–1.17)	0.738	---	---
Rural	0.74 (0.45–1.22)	0.240	---	---
Unknown	0.92 (0.66–1.28)	0.632	---	---
**Facility Location**				
East	1		1	
South	0.97 (0.81–1.18)	0.779	1.06 (0.86–1.31)	0.589
Central	1.05 (0.86–1.28)	0.617	1.09 (0.88–1.36)	0.422
West	1.23 (0.98–1.55)	0.077	1.32 (1.03–1.70)	0.031
Unknown	1.38 (1.13–1.70)	0.002	1.36 (1.04–1.78)	0.027
**Facility Type**				
Non-Academic	1		1	
Academic	1.35 (1.18–1.55)	<0.001	1.17 (1.00–1.37)	0.052
Unknown	1.56 (1.31–1.85)	<0.001		
**Insurance**				
Commercial	1		1	
Medicare	0.71 (0.62–0.83)	<0.001	0.78 (0.64–0.95)	0.012
Medicaid	1.03 (0.85–1.26)	0.761	0.86 (0.69–1.06)	0.161
Uninsured	0.75 (0.57–0.98)	0.035	0.66 (0.49–0.88)	0.005
Other	0.83 (0.57–1.21)	0.342	0.80 (0.53–1.20)	0.279
**Distance to Treatment Facility**				
≤40 miles	1		1	
>40 miles	1.20 (1.03–1.39)	0.018	0.97 (0.83–1.15)	0.748
Unknown	0.89 (0.54–1.47)	0.646	0.67 (0.39–1.14)	0.139
**Zip Code Education Level**				
≥21%	1		---	---
13–20.9%	1.13 (0.93–1.36)	0.211	---	---
7–12.9%	1.10 (0.92–1.32)	0.310	---	---
<7%	1.02 (0.84–1.23)	0.854	---	---
Unknown	0.95 (0.57–1.58)	0.839	---	---
**Zip Code Income Level**				
<38,000	1		---	---
38,000–47,999	1.05 (0.86–1.27)	0.653	---	---
48,000–62,999	1.10 (0.91–1.32)	0.330	---	---
≥63,000	1.03 (0.86–1.23)	0.774	---	---
Unknown	0.92 (0.56–1.52)	0.745	---	---
**Charlson Deyo Score**				
0	1		---	---
1	0.91 (0.76–1.08)	0.286	---	---
2	0.94 (0.65–1.35)	0.740	---	---
3	0.56 (0.27–1.18)	0.128	---	---
**Primary Site**				
Extremity	1		1	
Head and Neck	0.88 (0.63–1.23)	0.458	1.13 (0.79–1.63)	0.504
Thorax	0.50 (0.41–0.61)	<0.001	0.55 (0.44–0.69)	<0.001
Abdomen/Pelvis	0.44 (0.38–0.51)	<0.001	0.49 (0.42–0.57)	<0.001
Other/NOS	0.18 (0.14–0.23)	<0.001	0.31 (0.23–0.40)	<0.001
**Histology**				
Unclassified	1		1	
Undifferentiated Pleomorphic	1.73 (1.28–2.33)	<0.001	1.64 (1.18–2.26)	0.003
Fibrosarcoma/Myxofibrosarcoma	2.11 (1.50–2.97)	<0.001	2.06 (1.42–2.97)	<0.001
Liposarcoma	1.42 (1.11–1.81)	0.005	1.49 (1.14–1.93)	0.003
Leiomyosarcoma	0.78 (0.66–0.92)	0.003	1.09 (0.90–1.31)	0.379
Synovial Sarcoma	1.81 (1.44–2.28)	<0.001	1.67 (1.30–2.14)	<0.001
Angiosarcoma	0.66 (0.51–0.84)	0.001	1.00 (0.76–1.32)	0.995
MPNST	1.79 (1.29–2.47)	<0.001	1.97 (1.39–2.80)	<0.001
**Tumor Size**				
<5 cm	1		1	
5.1–10 cm	0.94 (0.75–1.18)	0.590	0.85 (0.67–1.08)	0.191
10.1–15 cm	1.01 (0.80–1.28)	0.928	0.87 (0.68–1.12)	0.280
>15 cm	0.96 (0.76–1.21)	0.727	0.80 (0.62–1.03)	0.088
Unknown	0.33 (0.26–0.42)	<0.001	0.41 (0.32–0.53)	<0.001
**Grade**				
I	1		1	
II	1.17 (0.69–1.99)	0.566	1.08 (0.61–1.90)	0.800
III	1.27 (0.79–2.03)	0.322	1.15 (0.69–1.90)	0.592
Unknown	0.54 (0.33–0.86)	0.010	0.55 (0.33–0.91)	0.020
**Year of Diagnosis**				
2004–2007	1		1	
2008–2011	0.98 (0.83–1.17)	0.852	1.01 (0.84–1.22)	0.913
2012–2015	0.85 (0.72–1.01)	0.060	0.92 (0.77–1.11)	0.389

**Table 2:** Survival odds in patients with metastatic STS. Univariate, multivariate, and propensity score matched analyses, respectively.

	HR (95% CI)	*p* Value	HR (95% CI)	*p* Value	HR (95% CI)	*p* Value
**Local Therapy**						
None	1		1		1	
Radiation Alone	0.79 (0.70–0.90)	<0.001	0.77 (0.67–0.87)	<0.001	0.75 (0.66–0.86)	<0.001
Surgery Alone	0.63 (0.58–0.69)	<0.001	0.67 (0.61–0.73)	<0.001	0.66 (0.60–0.72)	<0.001
Surgery and Radiation	0.41 (0.36–0.48)	<0.001	0.42 (0.36–0.48)	<0.001	0.41 (0.35–0.47)	<0.001
**Age**						
<70 years	1		1		---	---
≥70 years	1.35 (1.24–1.48)	<0.001	1.15 (1.02–1.29)	0.023	---	---
**Gender**						
Male	1		1		---	---
Female	0.87 (0.81–0.93)	<0.001	0.90 (0.83–0.96)	0.003	---	---
**Race**						
Non-Hispanic White	1		1		---	---
Non-Hispanic Black	0.92 (0.83–1.02)	0.106	0.92 (0.82–1.03)	0.149	---	---
Hispanic	0.68 (0.59–0.79)	<0.001	0.73 (0.63–0.86)	<0.001	---	---
Other	0.88 (0.73–1.04)	0.138	0.89 (0.74–1.07)	0.210	---	---
**Facility Area**						
Metropolitan	1		1		---	---
Urban	1.17 (1.05–1.30)	0.003	1.12 (1.00–1.27)	0.053	---	---
Rural	1.14 (0.86–1.51)	0.351	1.18 (0.88–1.59)	0.263	---	---
Unknown	1.27 (1.06–1.52)	0.010	1.05 (0.83–1.31)	0.704	---	---
**Facility Location**						
East	1		1		---	---
South	0.99 (0.89–1.10)	0.889	0.92 (0.82–1.04)	0.179	---	---
Central	1.04 (0.93–1.16)	0.498	0.99 (0.88–1.11)	0.852	---	---
West	0.90 (0.79–1.03)	0.130	0.87 (0.76–1.01)	0.060	---	---
Unknown	0.75 (0.67–0.84)	<0.001	0.68 (0.59–0.79)	<0.001	---	---
**Facility Type**						
Non-Academic	1		1		---	---
Academic	0.82 (0.76–0.89)	<0.001	0.84 (0.77–0.91)	<0.001	---	---
Unknown	0.68 (0.62–0.75)	<0.001			---	---
**Insurance**						
Commercial	1		1		---	---
Medicare	1.34 (1.24–1.46)	<0.001	1.13 (1.01–1.25)	0.026	---	---
Medicaid	0.96 (0.85–1.08)	0.498	1.01 (0.89–1.14)	0.891	---	---
Uninsured	1.03 (0.88–1.20)	0.725	1.05 (0.90–1.24)	0.520	---	---
Other	1.06 (0.86–1.32)	0.563	1.05 (0.84–1.30)	0.678	---	---
**Distance to Treatment Facility**						
≤40 miles	1		1		---	---
>40 miles	0.91 (0.84–0.99)	0.036	0.93 (0.84–1.03)	0.157	---	---
Unknown	1.50 (1.15–1.96)	0.003	1.75 (0.59–5.17)	0.309	---	---
**Zip Code Education Level**						
≥21%	1		1		---	---
13–20.9%	1.09 (0.98–1.21)	0.122	1.06 (0.95–1.19)	0.304	---	---
7–12.9%	1.09 (0.98–1.21)	0.097	1.05 (0.93–1.20)	0.423	---	---
<7%	1.03 (0.92–1.15)	0.626	1.02 (0.88–1.19)	0.757	---	---
Unknown	1.55 (1.18–2.04)	0.002	0.57 (0.12–2.74)	0.482	---	---
**Zip Code Income Level**						
<38,000	1		1		---	---
38,000–47,999	1.02 (0.92–1.14)	0.673	1.01 (0.89–1.14)	0.902	---	---
48,000–62,999	1.02 (0.92–1.14)	0.708	0.95 (0.84–1.08)	0.444	---	---
≥63,000	0.94 (0.85–1.04)	0.240	0.89 (0.76–1.03)	0.115	---	---
Unknown	1.47 (1.12–1.92)	0.005	1.41 (0.44–4.46)	0.564	---	---
**Charlson Deyo Score**						
0	1		1		---	---
1	1.16 (1.05–1.29)	0.003	1.08 (0.97–1.20)	0.151	---	---
2	1.56 (1.27–1.92)	<0.001	1.39 (1.12–1.72)	0.003	---	---
3	2.27 (1.52–3.39)	<0.001	2.48 (1.64–3.74)	<0.001	---	---
**Primary Site**						
Head and Neck	1		1		---	---
Upper Extremity	0.78 (0.62–0.98)	0.034	0.82 (0.65–1.04)	0.105	---	---
Lower Extremity	0.75 (0.62–0.91)	0.003	0.78 (0.64–0.95)	0.014	---	---
Thorax	1.12 (0.91–1.38)	0.281	1.02 (0.83–1.26)	0.831	---	---
Abdomen/Pelvis	0.94 (0.77–1.13)	0.495	0.93 (0.76–1.13)	0.478	---	---
Other/NOS	0.98 (0.79–1.20)	0.813	0.81 (0.65–1.00)	0.050	---	---
**Histology**						
Unclassified	1		1		---	---
Undifferentiated Pleomorphic	0.91 (0.77–1.07)	0.247	0.91 (0.76–1.07)	0.259	---	---
Fibrosarcoma/Myxofibrosarcoma	0.66 (0.53–0.82)	<0.001	0.78 (0.63–0.98)	0.031	---	---
Liposarcoma	0.79 (0.68–0.91)	0.001	0.86 (0.74–1.00)	0.050	---	---
Leiomyosarcoma	0.68 (0.62–0.75)	<0.001	0.63 (0.57–0.69)	<0.001	---	---
Synovial Sarcoma	0.69 (0.61–0.79)	<0.001	0.89 (0.77–1.02)	0.092	---	---
Angiosarcoma	1.12 (0.98–1.29)	0.094	1.06 (0.92–1.22)	0.440	---	---
MPNST	1.00 (0.84–1.20)	0.976	1.20 (1.00–1.45)	0.052	---	---
**Tumor Size**						
<5 cm	1		1		---	---
5.1–10 cm	1.13 (0.98–1.29)	0.083	1.18 (1.03–1.36)	0.017	---	---
10.1–15 cm	1.15 (1.00–1.31)	0.054	1.23 (1.06–1.42)	0.005	---	---
>15 cm	1.16 (1.01–1.33)	0.033	1.29 (1.12–1.49)	0.001	---	---
Unknown	1.43 (1.25–1.63)	<0.001	1.31 (1.14–1.50)	<0.001	---	---
**Grade**						
I	1		1		---	---
II	1.37 (0.98–1.92)	0.068	1.48 (1.05–2.08)	0.025	---	---
III	1.93 (1.43–2.61)	<0.001	1.98 (1.45–2.69)	<0.001	---	---
Unknown	1.86 (1.37–2.51)	<0.001	1.74 (1.28–2.37)	<0.001	---	---
**Year of Diagnosis**						
2004–2007	1		1		---	---
2008–2011	0.90 (0.83–0.99)	0.026	0.90 (0.82–0.98)	0.021	---	---
2012–2015	0.90 (0.82–0.99)	0.023	0.89 (0.80–0.98)	0.014	---	---

## Data Availability

The dataset was obtained from the National Cancer Database. It is available to COC-accredited institutions upon request, as detailed at https://www.facs.org/quality-programs/cancer/ncdb/puf (accessed on 9 January 2021).

## Supplementary Materials

The following are available online at https://www.mdpi.com/2072-6694/13/5/932/s1, Table S1: Baseline characteristics of patient cohort, Table S2: Factors associated with receipt of surgery as definitive local treatment, Table S3: Factors associated with receipt of radiotherapy as definitive local treatment, Table S4: Factors associated with receipt of surgery and radiotherapy as definitive local treatment.
