# Supplementary material for: Definitive Local Therapy Is Associated with Improved Survival in Metastatic Soft Tissue Sarcomas
Source: Cancers (Basel). 2021 Feb 24;13(5):932. doi: 10.3390/cancers13050932 (PMC7956624; doi:10.3390/cancers13050932)
Supplement: Supplementary file 1 [file cancers-13-00932-s001.pdf]

cancers

MDPI

Supplementary Materials:

Supplemental Table 1: Baseline characteristics of patient cohort.

**Receipt of local therapy**	**No**	%	**Yes**	%	**Total**	%	χ^2
**Total, n**	2,466	*59*	1,714	*41*	4,180	*100*	
**Age**							<0.001
<70 years	1,967	80	1,443	*84*	3,410	*82*	
≥70 years	499	20	271	*16*	770	*18*	
**Gender**							0.853
Male	1,351	*55*	944	*55*	2,295	*55*	
Female	1,115	*45*	770	*45*	1,885	*45*	
**Race**							0.736
Non-Hispanic White	1,761	*71*	1,250	*73*	3,011	*72*	
Non-Hispanic Black	381	*15*	246	*14*	627	*15*	
Hispanic	208	*8*	140	*8*	348	*8*	
Other	116	*5*	78	*5*	194	*5*	
**Facility Area**							0.649
Metropolitan	2,009	*81*	1,413	*82*	3,422	*82*	
Urban	317	*13*	216	*13*	533	*13*	
Rural	46	*2*	24	*1*	70	*2*	
Unknown	94	*4*	61	*4*	155	*4*	
**Insurance**							<0.001
Commercial	1,203	*49*	935	*55*	2,138	*51*	
Medicare	760	*31*	422	*25*	1,182	*28*	
Medicaid	272	*11*	218	*13*	490	*12*	
Uninsured	157	*6*	91	*5*	248	*6*	
Other	74	*3*	48	*3*	122	*3*	

**Zip Code Education Level**							0.632
≥21%	472	*19*	308	*18*	780	*19*	
13%-20.9%	613	*25*	451	*26*	1,064	*25*	
7%-12.9%	752	*30*	539	*31*	1,291	*31*	
<7%	587	*24*	390	*23*	977	*23*	
Unknown	42	*2*	26	*2*	68	*2*	
**Zip Code Income Level**							0.851
<38,000	459	*19*	306	*18*	765	*18*	
38,000-47,999	574	*23*	400	*23*	974	*23*	
48,000-62,999	634	*26*	464	*27*	1,098	*26*	
≥63,000	755	*31*	517	*30*	1,272	*30*	
Unknown	44	*2*	27	*2*	71	*2*	
**Facility Type**							<0.001
Non-Academic	1,075	*44*	606	*35*	1,681	*40*	
Academic	967	*39*	736	*43*	1,703	*41*	
Unknown	424	*17*	372	*22*	796	*19*	
**Facility Location**							0.001
East	445	*18*	282	*16*	727	*17*	
South	749	*30*	462	*27*	1,211	*29*	
Central	558	*23*	372	*22*	930	*22*	
West	290	*12*	226	*13*	516	*12*	
Unknown	424	*17*	372	*22*	796	*19*	
**Distance to Treatment Facility**							0.050
≤40 miles	1,917	*78*	1,283	*75*	3,200	*77*	
>40 miles	507	*21*	406	*24*	913	*22*	
Unknown/Missing	42	*2*	25	*1*	67	*2*	
**Charlson Deyo Score**							0.326

0	2,001	*81*	1,419	*83*	3,420	*82*	
1	365	*15*	235	*14*	600	*14*	
2	75	*3*	50	*3*	125	*3*	
3	25	*1*	10	*1*	35	*1*	
**Primary Site**							<0.001
Head and Neck	72	*3*	77	*4*	149	*4*	
Extremity	693	*28*	842	*49*	1,535	*37*	
Thorax	318	*13*	193	*11*	511	*12*	
Abdomen/Pelvis	955	*39*	508	*30*	1,463	*35*	
Other/NOS	428	*17*	94	*5*	522	*12*	
**Histology**							<0.001
Unclassified	1,051	*43*	682	*40*	1,733	*41*	
Undifferentiated Pleomorphic	90	*4*	101	*6*	191	*5*	
Fibrosarcoma/Myxofibrosarcoma	62	*3*	85	*5*	147	*4*	
Liposarcoma	162	*7*	149	*9*	311	*7*	
Leiomyosarcoma	626	*25*	316	*18*	942	*23*	
Synovial Sarcoma	165	*7*	194	*11*	359	*9*	
Angiosarcoma	235	*10*	100	*6*	335	*8*	
MPNST	75	*3*	87	*5*	162	*4*	
**Grade**							<0.001
I	41	*2*	32	*2*	73	*2*	
II	114	*5*	104	*6*	218	*5*	
III	1,071	*43*	1,060	*62*	2,131	*51*	
Unknown	1,240	*50*	518	*30*	1,758	*42*	
**Tumor Size**							<0.001
<5 cm	225	*9*	207	*12*	432	*10*	
5.1-10 cm	547	*22*	473	*28*	1,020	*24*	

10.1-15 cm	414	*17*	385	*22*	799	*19*	
>15 cm	452	*18*	399	*23*	851	*20*	
Unknown	828	*34*	250	*15*	1,078	*26*	
**Year of Diagnosis**							0.064
2004-2007	477	*19*	358	*21*	835	*20*	
2008-2011	837	*34*	618	*36*	1,455	*35*	
2012-2015	1,152	*47*	738	*43*	1,890	*45*	

Supplemental Table 2: Factors associated with receipt of surgery as definitive local treatment.

	Univariate Analysis			Multivariate Analysis	
**Receipt of surgery**	**OR (95% CI)**	***P* value**		**OR (95% CI)**	***P* value**
**Age**					
<70 years	1			1	
≥70 years	0.65 (0.53-0.79)	<0.001		0.75 (0.58-0.97)	0.026
**Gender**					
Male	1			---	---
Female	1.07 (0.93-1.23)	0.355		---	---
**Race**					
Non-Hispanic White	1			---	---
Non-Hispanic Black	0.92 (0.75-1.13)	0.445		---	---
Hispanic	1.04 (0.81-1.35)	0.741		---	---
Other	0.96 (0.68-1.36)	0.821		---	---
**Facility Area**					
Metropolitan	1			---	---
Urban	0.90 (0.73-1.12)	0.365		---	---
Rural	1.02 (0.59-1.77)	0.945		---	---
Unknown	1.03 (0.71-1.50)	0.868		---	---
**Facility Location**					

East	1			1	
South	1.01 (0.81-1.25)	0.962		1.09 (0.85-1.38)	0.501
Central	0.89 (0.71-1.13)	0.348		0.90 (0.70-1.16)	0.414
West	1.16 (0.89-1.50)	0.280		1.22 (0.92-1.62)	0.158
Unknown	1.22 (0.96-1.53)	0.101		1.22 (0.90-1.65)	0.202
**Facility Type**					
Non-Academic	1			1	
Academic	1.38 (1.17-1.62)	<0.001		1.21 (1.01-1.45)	0.037
Unknown	1.45 (1.19-1.76)	<0.001		.	.
**Insurance**					
Commercial	1			1	
Medicare	0.69 (0.58-0.83)	<0.001		0.82 (0.66-1.02)	0.079
Medicaid	1.12 (0.90-1.40)	0.313		1.03 (0.82-1.31)	0.778
Uninsured	0.77 (0.56-1.07)	0.117		0.74 (0.53-1.04)	0.084
Other	0.99 (0.65-1.51)	0.975		1.01 (0.65-1.57)	0.953
**Distance to Treatment Facility**					
≤40 miles	1			1	
>40 miles	1.33 (1.13-1.57)	0.001		1.11 (0.92-1.32)	0.274
Unknown	0.99 (0.55-1.77)	0.973		0.79 (0.43-1.45)	0.451
**Zip Code Education Level**					
≥21%	1			---	---
13%-20.9%	1.07 (0.86-1.34)	0.517		---	---
7%-12.9%	1.09 (0.88-1.34)	0.429		---	---
<7%	0.99 (0.79-1.24)	0.951		---	---
Unknown	1.03 (0.58-1.85)	0.913		---	---
**Zip Code Income Level**					
<38,000	1			---	---
38,000-47,999	1.08 (0.86-1.35)	0.522		---	---

48,000-62,999	1.07 (0.86-1.33)	0.531		---	---
≥63,000	1.09 (0.88-1.35)	0.411		---	---
Unknown	1.00 (0.56-1.78)	0.988		---	---
**Charlson Deyo Score**					
0	1			---	---
1	1.00 (0.82-1.23)	0.995		---	---
2	1.26 (0.84-1.87)	0.264		---	---
3	0.67 (0.28-1.61)	0.370		---	---
**Primary Site**					
Extremity	1			1	
Head and Neck	0.95 (0.66-1.38)	0.803		1.10 (0.74-1.63)	0.652
Thorax	0.68 (0.54-0.86)	0.001		0.75 (0.59-0.97)	0.027
Abdomen/Pelvis	0.67 (0.56-0.79)	<0.001		0.73 (0.61-0.88)	0.001
Other/NOS	0.27 (0.20-0.36)	<0.001		0.43 (0.31-0.59)	<0.001
**Histology**					
Unclassified	1			1	
Undifferentiated Pleomorphic	1.77 (1.28-2.45)	0.001		1.72 (1.22-2.42)	0.002
Fibrosarcoma/Myxofibrosarcoma	2.19 (1.54-3.12)	<0.001		2.07 (1.42-3.01)	<0.001
Liposarcoma	1.29 (0.97-1.70)	0.079		1.31 (0.97-1.76)	0.075
Leiomyosarcoma	1.08 (0.89-1.31)	0.418		1.42 (1.15-1.75)	0.001
Synovial Sarcoma	1.67 (1.30-2.15)	<0.001		1.59 (1.21-2.09)	0.001
Angiosarcoma	0.76 (0.56-1.03)	0.079		1.07 (0.77-1.50)	0.676
MPNST	2.16 (1.54-3.04)	<0.001		2.31 (1.61-3.31)	<0.001
**Tumor Size**					
<5 cm	1			1	
5.1-10 cm	0.68 (0.53-0.87)	0.002		0.61 (0.47-0.79)	<0.001
10.1-15 cm	0.76 (0.59-0.98)	0.033		0.65 (0.50-0.86)	0.002
>15 cm	0.78 (0.60-1.00)	0.047		0.66 (0.51-0.86)	0.003

Unknown	0.30 (0.23-0.39)	<0.001		0.37 (0.28-0.49)	<0.001
**Grade**					
I	1			1	
II	0.86 (0.47-1.55)	0.606		0.82 (0.45-1.52)	0.536
III	1.07 (0.64-1.79)	0.797		1.08 (0.63-1.85)	0.780
Unknown	0.45 (0.27-0.76)	0.003		0.49 (0.29-0.85)	0.011
**Year of Diagnosis**					
2004-2007	1			---	---
2008-2011	1.10 (0.90-1.34)	0.357		---	---
2012-2015	0.99 (0.81-1.20)	0.901		---	---

Supplemental Table 3: Factors associated with receipt of radiotherapy as definitive local treatment.

	**Univariate Analysis**			**Multivariate Analysis**	
**Receipt of radiotherapy**	**OR (95% CI)**	***P* value**		**OR (95% CI)**	***P* value**
**Age**					
<70 years	1			1	
≥70 years	1.26 (0.98-1.63)	0.076		1.28 (0.99-1.67)	0.064
**Gender**					
Male	1			---	---
Female	0.85 (0.69-1.05)	0.136		---	---
**Race**					
Non-Hispanic White	1			---	---
Non-Hispanic Black	0.88 (0.65-1.20)	0.427		---	---
Hispanic	0.71 (0.46-1.09)	0.118		---	---
Other	1.04 (0.64-1.69)	0.880		---	---
**Facility Area**					
Metropolitan	1			---	---
Urban	0.95 (0.69-1.31)	0.741		---	---

Rural	0.59 (0.21-1.64)	0.314		---	---
Unknown	0.90 (0.50-1.60)	0.712		---	---
**Facility Location**					
East	1			---	---
South	0.96 (0.69-1.33)	0.821		---	---
Central	1.07 (0.76-1.50)	0.699		---	---
West	1.21 (0.83-1.77)	0.325		---	---
Unknown	1.05 (0.74-1.49)	0.802		---	---
**Facility Type**					
Non-Academic	1			---	---
Academic	0.94 (0.75-1.19)	0.632		---	---
Unknown	0.98 (0.73-1.31)	0.892		---	---
**Insurance**					
Commercial	1			---	---
Medicare	1.07 (0.84-1.36)	0.604		---	---
Medicaid	1.06 (0.75-1.48)	0.752		---	---
Uninsured	0.94 (0.59-1.51)	0.807		---	---
Other	1.01 (0.53-1.91)	0.975		---	---
**Distance to Treatment Facility**					
≤40 miles	1			1	
>40 miles	0.60 (0.45-0.81)	0.001		0.60 (0.45-0.81)	0.001
Unknown	0.89 (0.38-2.08)	0.790		0.87 (0.37-2.03)	0.740
**Zip Code Education Level**					
≥21%	1			---	---
13%-20.9%	1.19 (0.86-1.65)	0.290		---	---
7%-12.9%	1.09 (0.79-1.49)	0.611		---	---
<7%	1.12 (0.80-1.56)	0.519		---	---

Unknown	1.06 (0.44-2.56)	0.889		---	---
**Zip Code Income Level**					
<38,000	1			---	---
38,000-47,999	1.06 (0.75-1.48)	0.750		---	---
48,000-62,999	1.14 (0.82-1.57)	0.432		---	---
≥63,000	1.10 (0.80-1.51)	0.551		---	---
Unknown	0.99 (0.41-2.38)	0.989		---	---
**Charlson Deyo Score**					
0	1			---	---
1	0.80 (0.58-1.10)	0.165		---	---
2	0.66 (0.32-1.35)	0.254		---	---
3	1.24 (0.43-3.53)	0.690		---	---
**Primary Site**					
Extremity	1			1	
Head and Neck	1.22 (0.73-2.06)	0.447		1.37 (0.80-2.35)	0.251
Thorax	0.99 (0.71-1.38)	0.939		1.00 (0.71-1.42)	0.988
Abdomen/Pelvis	0.82 (0.64-1.05)	0.111		0.90 (0.69-1.16)	0.402
Other/NOS	0.62 (0.42-0.91)	0.015		0.70 (0.46-1.05)	0.085
**Histology**					
Unclassified	1			1	
Undifferentiated Pleomorphic	0.71 (0.41-1.23)	0.219		0.67 (0.39-1.17)	0.162
Fibrosarcoma/Myxofibrosarcoma	0.67 (0.36-1.27)	0.220		0.68 (0.36-1.29)	0.243
Liposarcoma	0.92 (0.62-1.38)	0.687		0.95 (0.63-1.43)	0.799
Leiomyosarcoma	0.58 (0.43-0.78)	<0.001		0.61 (0.44-0.82)	0.001
Synovial Sarcoma	0.96 (0.66-1.39)	0.812		0.97 (0.66-1.41)	0.863
Angiosarcoma	0.79 (0.52-1.19)	0.255		0.83 (0.54-1.27)	0.398
MPNST	0.61 (0.32-1.14)	0.119		0.66 (0.35-1.25)	0.205

**Tumor Size**					
<5 cm	1			1	
5.1-10 cm	1.60 (1.05-2.44)	0.028		1.62 (1.06-2.48)	0.027
10.1-15 cm	1.51 (0.98-2.34)	0.063		1.56 (1.00-2.43)	0.052
>15 cm	1.31 (0.85-2.04)	0.223		1.40 (0.89-2.20)	0.145
Unknown	1.15 (0.74-1.77)	0.534		1.24 (0.80-1.93)	0.337
**Grade**					
I	1			---	---
II	2.23 (0.75-6.65)	0.149		---	---
III	1.80 (0.65-4.97)	0.260		---	---
Unknown	1.62 (0.58-4.50)	0.354		---	---
**Year of Diagnosis**					
2004-2007	1			---	---
2008-2011	0.85 (0.64-1.14)	0.273		---	---
2012-2015	0.86 (0.65-1.13)	0.285		---	---

Supplemental Table 4: Factors associated with receipt of surgery and radiotherapy as definitive local treatment.

	**Univariate Analysis**			**Multivariate Analysis**	
**Receipt of Surgery + RT**	**OR (95% CI)**	***P* value**		**OR (95% CI)**	***P* value**
**Age**					
<70 years	1			1	
≥70 years	0.76 (0.56-1.04)	0.086		0.92 (0.62-1.38)	0.698
**Gender**					
Male	1			---	---
Female	0.98 (0.78-1.22)	0.840		---	---
**Race**					
Non-Hispanic White	1			---	---
Non-Hispanic Black	1.02 (0.75-1.40)	0.896		---	---

Hispanic	1.07 (0.72-1.58)	0.755		---	---
Other	0.88 (0.50-1.54)	0.649		---	---
**Facility Area**					
Metropolitan	1			---	---
Urban	1.20 (0.88-1.64)	0.252		---	---
Rural	0.51 (0.16-1.63)	0.254		---	---
Unknown	0.78 (0.41-1.50)	0.462		---	---
**Facility Location**					
East	1			1	
South	0.93 (0.65-1.35)	0.708		0.97 (0.65-1.44)	0.873
Central	1.42 (0.99-2.03)	0.060		1.45 (0.99-2.13)	0.057
West	1.11 (0.72-1.71)	0.647		1.13 (0.71-1.79)	0.606
Unknown	1.64 (1.14-2.36)	0.008		1.54 (0.96-2.48)	0.075
**Facility Type**					
Non-Academic	1			1	
Academic	1.33 (1.03-1.72)	0.030		1.04 (0.78-1.38)	0.783
Unknown	1.73 (1.29-2.33)	<0.001		.	.
**Insurance**					
Commercial	1			1	
Medicare	0.74 (0.57-0.97)	0.027		0.87 (0.61-1.22)	0.412
Medicaid	0.78 (0.54-1.12)	0.181		0.59 (0.40-0.86)	0.007
Uninsured	0.77 (0.46-1.27)	0.299		0.66 (0.39-1.12)	0.123
Other	0.51 (0.22-1.17)	0.110		0.46 (0.19-1.07)	0.072
**Distance to Treatment Facility**					
≤40 miles	1			1	
>40 miles	1.40 (1.09-1.79)	0.009		1.19 (0.91-1.56)	0.212
Unknown	0.77 (0.28-2.14)	0.620		0.59 (0.21-1.70)	0.332

**Zip Code Education Level**					
≥21%	1			---	---
13%-20.9%	1.02 (0.73-1.43)	0.902		---	---
7%-12.9%	1.01 (0.73-1.40)	0.947		---	---
<7%	0.96 (0.68-1.35)	0.804		---	---
Unknown	0.70 (0.25-1.98)	0.501		---	---
**Zip Code Income Level**					
<38,000	1			---	---
38,000-47,999	0.92 (0.65-1.29)	0.616		---	---
48,000-62,999	0.99 (0.72-1.37)	0.967		---	---
≥63,000	0.79 (0.57-1.10)	0.158		---	---
Unknown	0.78 (0.30-1.99)	0.599		---	---
**Charlson Deyo Score**					
0	1			---	---
1	0.93 (0.67-1.28)	0.648		---	---
2	0.65 (0.30-1.40)	0.270		---	---
3	.	.		---	---
**Primary Site**					
Extremity	1			1	
Head and Neck	0.69 (0.40-1.17)	0.169		0.95 (0.54-1.67)	0.845
Thorax	0.32 (0.21-0.48)	<0.001		0.37 (0.24-0.57)	<0.001
Abdomen/Pelvis	0.25 (0.19-0.34)	<0.001		0.29 (0.21-0.39)	<0.001
Other/NOS	0.08 (0.04-0.17)	<0.001		0.15 (0.07-0.32)	<0.001
**Histology**					
Unclassified	1			1	
Undifferentiated Pleomorphic	1.81 (1.15-2.86)	0.011		1.58 (0.98-2.55)	0.062
Fibrosarcoma/Myxofibrosarcoma	1.89 (1.14-3.13)	0.013		1.64 (0.97-2.79)	0.067

Liposarcoma	1.72 (1.18-2.52)	0.005		1.80 (1.20-2.70)	0.005
Leiomyosarcoma	0.60 (0.43-0.85)	0.004		0.89 (0.61-1.29)	0.544
Synovial Sarcoma	1.81 (1.27-2.58)	0.001		1.38 (0.94-2.02)	0.098
Angiosarcoma	0.56 (0.33-0.97)	0.040		0.96 (0.54-1.71)	0.883
MPNST	1.41 (0.83-2.40)	0.206		1.35 (0.77-2.37)	0.299
**Tumor Size**					
<5 cm	1			1	
5.1-10 cm	1.37 (0.92-2.05)	0.125		1.30 (0.85-1.98)	0.225
10.1-15 cm	1.41 (0.93-2.14)	0.104		1.23 (0.79-1.90)	0.359
>15 cm	1.33 (0.88-2.02)	0.174		1.07 (0.69-1.67)	0.753
Unknown	0.30 (0.18-0.50)	<0.001		0.42 (0.25-0.72)	0.001
**Grade**					
I	1			1	
II	1.11 (0.46-2.71)	0.815		1.00 (0.39-2.57)	0.999
III	1.06 (0.48-2.35)	0.879		0.91 (0.39-2.13)	0.831
Unknown	0.54 (0.24-1.21)	0.132		0.59 (0.25-1.39)	0.227
**Year of Diagnosis**					
2004-2007	1			1	
2008-2011	0.92 (0.68-1.23)	0.555		0.95 (0.70-1.29)	0.742
2012-2015	0.74 (0.55-0.98)	0.039		0.80 (0.59-1.09)	0.152
